# The New Cure for Consumption

**Published:** 1898-08

**Authors:** 


### EDITORIAL.

The office of THE JOURNAL is 305 and 306 Fitten Building.

Address all communications, and make all remittances payable to THE ATLANTA MEDICAL AND SURGICAL JOURNAL, Atlanta, Ga.

Reprints of articles will be furnished, when desired, at a small cost. Requests for the same should always be made *on the manuscript*.

### THE NEW CURE FOR CONSUMPTION.

The daily papers have been giving great space recently to a paper read by Dr. Jno. B. Murphy, of Chicago, before the last meeting of the American Medical Association. A paper coming from such a source and read by such a distinguished man and upon such an all-important subject was well calculated to attract attention. Dr. Murphy stands in the foremost ranks of American surgeons, a man of original ideas and a pioneer worker in all he undertakes. Under these circumstances his views on any subject will have great weight with the profession. We only trust that time and experience may verify the truthfulness of his theory—for such must it be until otherwise demonstrated. His theory certainly looks logical, if comparison is ever justifiable as a working basis. From what we can gather in the garbled medical extracts, the original paper having as yet not been published, Dr. Murphy's theory is this: In order for an abscess to heal, or even any inflamed tissue, the parts must be put thoroughly at rest, and this we always try to do. Now, the question is, if the lung in the tuberculous was put absolutely at rest, then this rest would give the tissue time to repair. Dr. Murphy proposes to accomplish this by filling the pleural cavity with nitrogen, and thus so compress the lung as to render its activity impossible. This gas can be easily removed whenever the condition of the patient demands it, and thus intervals of rest can be given to the diseased lung. We have not heard as yet whether other internal medication is given. We wait with a good deal of interest for the time when this treatment has been tried sufficiently long and thoroughly so as to give some trustworthy data upon which to base an opinion. New remedies for the cure of consumption are frequently brought forth; hence it is that the medical profession always looks to find out who the originator is. Dr. Murphy is too well known for his theory not to be given a full practical trial. The old adage, that "there is nothing new under the sun" certainly seems to be true. Just at this writing we find that Dr. W. H. Meyer, of Fort Wayne, comes forward with the statement that the "Murphy method," or "Chicago method," for treating phthisis is not new, and substantiates his statements by ample quotations from a recent work of Dr. Paget. He says: "In Rome, 1894, Professor C. Farlanini read notes of cases treated by forming an artificial pneumothorax. The whole method is set forth in vol. 3, Proceedings of the International Medical Congress for 1894. Farlanini stated the pleura bears air well, but that nitrogen is preferable, as it is absorbed more slowly. The therapeutic results were local, being overpowered by the general condition and that of the other affected lung. His paper went to show that the method only is practical where the lesion is limited and monolateral. The discussion was by Professors Teneo, of Pisa; Slone, of Bondeno; Sutherland, of North London Consumption Hospital. The latter thought the rest secured of great value. The same views were held by Hilton Flagg in 1891. In 1881 Dr. Herard, Hotel Dieu, Paris, stated that an accidental hydro-pneumothorax arrested phthisis and became an unhoped-for means of safety to the patient."

